# Family polyvictimization in households with parental health conditions: a cross-sectional study of disparities and risk factors

**DOI:** 10.3389/fpubh.2026.1892707

**Published:** 2026-08-14

**Authors:** Chengrui Wu, Qiqi Chen, Camilla K. M. Lo, Frederick Ho, Douglas Brownridge, Patrick Ip, Ko Ling Chan

**Affiliations:** 1 Department of Applied Social Science, The Hong Kong Polytechnic University, Hung Hom, Hong Kong SAR, China; 2 School of Health and Wellbeing, University of Glasgow, Glasgow, United Kingdom; 3 College of Global and Population Health, University of Manitoba, Winnipeg, MB, Canada; 4 Department of Paediatrics and Adolescent Medicine, The University of Hong Kong, Pokfulam, Hong Kong SAR, China

**Keywords:** caregiving burden, chronic illness, family polyvictimization, family violence, parents with disabilities

## Abstract

**Background:**

Families with parents with health conditions often experience socioeconomic hardship, caregiving strain, and elevated exposure to family violence. However, differences in family polyvictimization (FPV) between these families and those without health conditions remain underexplored. This study examined disparities in FPV exposure and associated family-level risk factors.

**Methods:**

A cross-sectional household survey was conducted with 1,400 Hong Kong families with children under 18. Measures included FPV (FPV Screen), social support (MSPSS), family hierarchy (PRP), health (SF-12v2, PedsQoL), and mental health (DASS-21). Logistic regression models were used to examine associations between family characteristics and having parents with health conditions, as well as differences in FPV exposure between the two family groups.

**Results:**

Families with parents with health conditions had significantly higher FPV prevalence, with elder victimization remaining significant after adjustment (aOR = 1.89, 95% CI: 1.08–3.29). These families also showed greater odds of co-occurring disadvantages, including caregiving responsibilities for children with special educational needs (aOR = 3.81) or older relatives (aOR = 1.71), lower household income (aOR = 1.71), reduced physical functioning (aOR = 0.93), limited social support (aOR = 0.83), and heightened exposure to violence socialization (aOR = 1.48).

**Conclusions:**

FPV is more prevalent in families with parents with health conditions, especially when combined with caregiving strain, economic disadvantage, and relational stress. These findings support integrating FPV screening and family-centered support into disability and caregiving services, with attention to elder victimization and low-social-support households.

## Introduction

Disability and chronic illness are significant public health issues affecting not only the individual, but also the broader family systems in which they live. Although these conditions differ in etiology, functional limitations, and illness trajectories, both are often associated with long-term health challenges that may disrupt family roles, increase caregiving demands, and heighten exposure to socioeconomic strain (1). In China, 17.8% of households report at least one family member with a disability (2). In 2020, approximately 1.80 million people in Hong Kong had chronic illnesses requiring long-term medical treatment, consultation, or medication, representing 24.1% of the total population (3). When disability or chronic illness occurs within a family, it often intersects with structural and socioeconomic disadvantage, complex caregiving demands, and heightened social stress (4). However, existing research has largely overlooked these family-level dynamics, focusing mainly on individual outcomes.

Importantly, these intersecting structural and caregiving pressures may not only affect wellbeing, but also increase vulnerability to violence within the household. Compared with typically developing peers, individuals with disabilities or chronic illnesses face higher and more diverse rates of victimization (5, 6). This risk may be especially pronounced when the family member with the health condition is a parent. In such families, balancing health-related challenges with caregiving responsibilities can increase stress, potentially affecting parenting capacity and also placing their children at greater risk of poor health and victimization (7).

Therefore, in families with parents with disabilities or chronic illness, vulnerability to violence may extend beyond the parents themselves to other household members, including children and older relatives, particularly in contexts of high caregiving burden and constrained resources. In such settings, violence may not occur as a single isolated event, but instead emerge as multiple, co-occurring forms of victimization within the same family system. Previous research has shown that families experiencing multiple types of violence reported significantly higher rates of parental PTSD and depressive symptoms (8). This underscores the need to examine violence as an interconnected phenomenon rather than isolated incidents. Although violence can take different forms, family victimization is commonly categorized as violence against children, violence between intimate partners, and violence against older adults (9).

Building on this framework, recent studies have introduced family polyvictimization (FPV), which refers to the co-occurrence of multiple types of violence within the same family (10). FPV differs from other uses of the term polyvictimization in the broader violence literature. Some studies define polyvictimization at the individual level, focusing on a single person who reports repeated incidents of family violence (11–13). Other studies use polyvictimization to describe individuals who experience multiple forms of victimization or trauma, including physical, psychological, sexual abuse, or neglect, where the perpetrators may not necessarily be family members (14–16). In both cases, the individual is treated as the primary unit of analysis.

In contrast, FPV conceptualizes the entire family as the primary unit of victimization. This perspective recognizes that family members' experiences are interconnected and that violence rarely occurs in isolation. Accordingly, FPV focuses on multiple victims within the same household, as well as the possibility that individuals may be both victims and perpetrators across different forms of violence. Prior research suggests that when one type of violence is detected in a family, other forms of violence are six times more likely to be present within the same family (17). Population-based evidence further indicates that 19.2% of Hong Kong families reported one to two victims, while 9.6% reported FPV within the same family (18).

However, not every family experiences FPV. Understanding why violence clusters in some families requires examining the shared factors that drive multiple forms of victimization. Different forms of violence within families often stem from shared underlying factors. Previous research has identified several risk factors contributing to the co-occurrence of multiple forms of violence, including acceptance of violent attitudes (19), social isolation (20), and broader family and environmental factors such as poverty, unemployment, parenting stress, and low socioeconomic status (21, 22). Importantly, many of these risk factors are disproportionally present in family with disabilities or chronic illnesses. These families are more likely to experience long-term health-related functional limitations, reduced labor market participation, increased financial strain and poverty (23, 24). Such conditions are strongly associated with heightened stress, limited coping resources, and increased dependence among family members (25), thereby increasing the risk of conflict, relational strain, and exposure to multiple forms of violence within the family system (4).

Despite these theoretically plausible pathways, FPV research has not directly compared families with vs. without parents with disabilities or chronic illness. Therefore, it remains unclear whether these families face a higher risk of FPV and more complex patterns of victimization, and how these patterns relate to the shared risk factors identified above. This gap is particularly important because families with parents with disabilities or chronic illness may be more visible to service systems, making them a potentially feasible entry point for early FPV screening and family-centered prevention.

## Current study

In this exploratory study, we examined families with parents with disabilities or chronic illness and assess the extent to which FPV co-occurs within these households. Because our focus is on these shared family-system factors rather than on the conditions themselves, we used the term “parental health conditions” in this study to refer collectively to parental chronic illness and disability, while acknowledging their heterogeneity. Families with health conditions were identified if they reported any condition that affected daily functioning. FPV was defined following prior research as the co-occurrence of multiple forms of family violence within the same family, including but not limited to violence against children, intimate partner violence, and violence against older adults (10). To operationalize this construct in the current study, a family was classified as experiencing FPV if three or more victims were reported, including a child, an adult parent, or an older family member (10, 18). This threshold was adopted to capture the presence of multi-person victimization within the family system, rather than isolated or dyadic incidents of violence. Because any single type of violence could involve at most two victims, as in bidirectional intimate partner violence, the identification of three or more victims necessarily indicated the co-occurrence of at least two distinct types of family violence. The criterion therefore distinguished FPV from isolated or exclusively dyadic incidents of violence. Specifically, we aimed to (1) compare FPV prevalence between families with and without parents with health conditions, and (2) describe how FPV clusters with key family vulnerabilities, including caregiving burden, family hierarchy, socioeconomic hardship, limited social support, and poorer health, among families with parents with health conditions. By using having parents with health conditions as a pragmatic starting point for identifying high-risk family configurations, this study seeks to strengthen the evidence base for integrating violence screening and family-level support into disability- and caregiving-related services.

## Methods

### Participants

**Figure 1:**
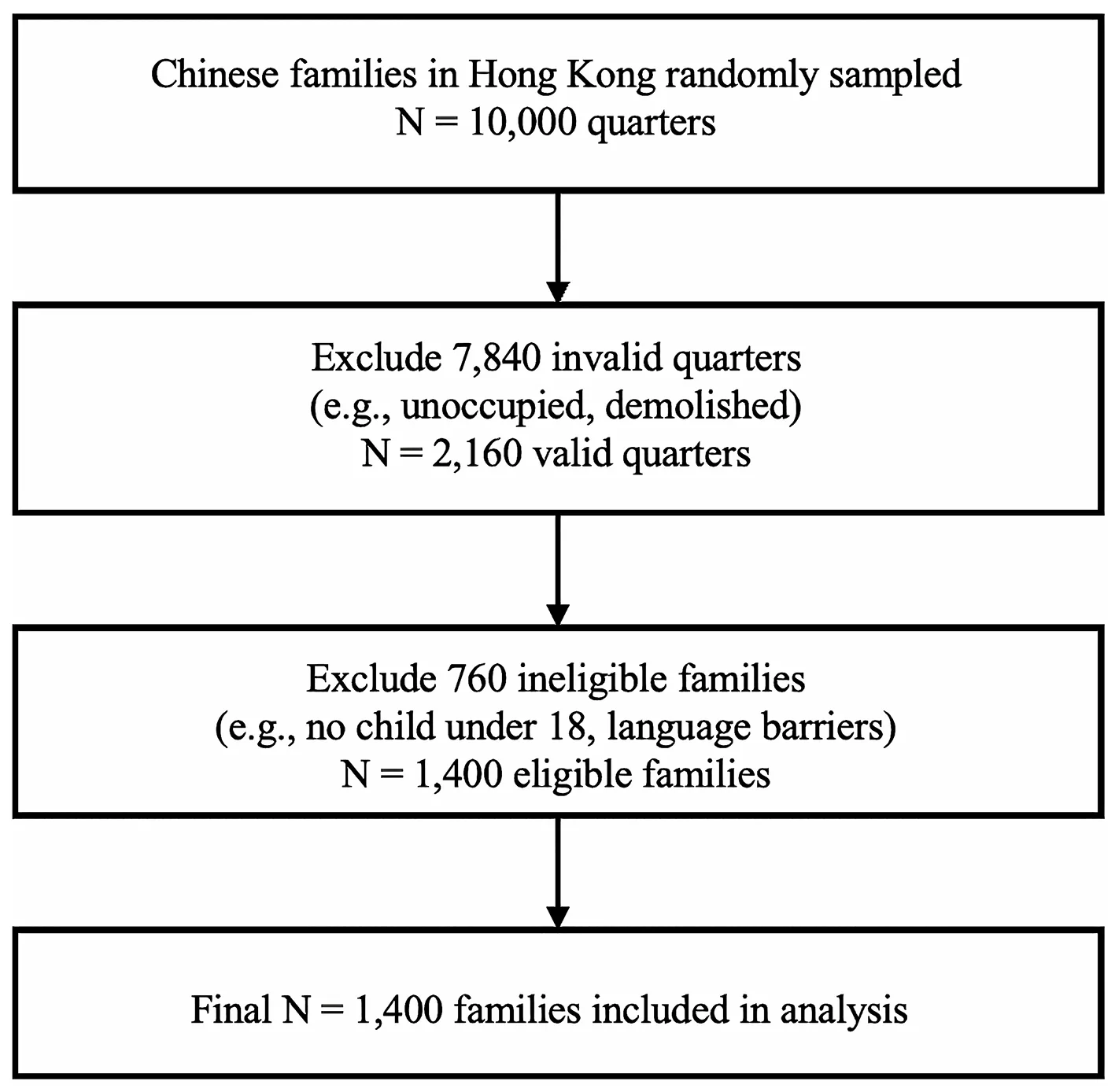
Sample selection process.

The target respondents included Chinese families living in Hong Kong with at least one child under 18 years of age. Participants had to be able to speak either Cantonese or Mandarin. From an initial sample of 10,000 households randomly sampled from the population, 2,160 valid households were identified after removing invalid cases such as unoccupied or demolished addresses. Ultimately, 1,400 eligible families completed the survey, resulting in a response rate of 64.8% (Figure 1).

### Procedure

This cross-sectional population-based household survey was conducted from August 2022 to March 2023 using a two-stage sampling approach. First, residential addresses stratified by geographic location and housing type were randomly selected from Hong Kong's Census and Statistics Department registry. Second, one eligible household from each selected address was randomly chosen. One parent from each selected household was randomly selected as the respondent. Data collection was conducted through face-to-face interviews administered by trained research assistants with prior household survey experience and specialized ethical training. Interviews were conducted in a private area within the household to ensure confidentiality. Responses were recorded directly into a secure, password-protected online survey platform using tablet devices provided by the research team. No personal identifiers were collected, and all data were transmitted through encrypted connections and stored on secure servers in accordance with data protection regulations. The study was conducted in accordance with the principles of the Declaration of Helsinki. Written informed consent was obtained from all participants prior to data collection. The study received ethical approval from the Ethics Committee of The Hong Kong Polytechnic University (Reference: HSEARS20191213002).

### Measures

#### Demographic characteristics

Respondents were asked to provide their demographic information, including co-residence with family members, age, gender, educational attainment, employment status, total family income, whether the family received Comprehensive Social Security Assistance (CSSA), and whether the family had members with health conditions. Parental health conditions were assessed using self-reported checklists for disability and chronic illness. Disability categories included physical disability, visual impairment, hearing impairment, speech impairment, mental illness, intellectual disability, autism, attention-deficit/hyperactivity disorder, and specific learning difficulties. For chronic illness, respondents could select multiple conditions from a predefined list comprising hypertension, heart disease, asthma, diabetes, kidney disease, cataract, tuberculosis, peptic ulcer, skin disease, and other chronic illnesses. Respondents were classified as having a parental health condition if they reported at least one disability or chronic illness.

#### Family polyvictimization

The Family Polyvictimization Screen (FPS) (26) assesses family violence across three modules: IPV self and partner's victimization (three items for each: physical, psychological, and sexual violence), child abuse and neglect (CAN) for all children (four items: physical, psychological, sexual violence, and neglect), and older adult(s) abuse and neglect (EAN) for all grandparents (five items: physical, psychological, and sexual violence, neglect and financial exploitation). Respondents indicate the perpetrator and timing (past year or earlier) for each dichotomous (yes/no) item. Victim–perpetrator relationships are used to categorize violence types, including IPV, in-law violence, parent or child abuse, and abuse by other relatives across age groups. Sample items include, “Have you ever experienced physical assault?” and “Has your child ever been neglected?” To help respondents answer accurately, standard examples of each type of violence are provided. Families are grouped into: (a) no victimization, (b) one or two victims, or (c) polyvictimization (three or more victims). The modules demonstrated satisfactory reliability in this study (α = 0.83–0.91).

#### Social support

The Multidimensional Scale of Perceived Social Support (MSPSS) (27) is 12-item instrument used to measure perceived social support from three sources: family, friends and a significant other. Each item is rated on a 7-point scale ranging from one (“very strongly disagree”) to seven (“very strongly agree”). A total score is calculated by summing responses for all 12 items. Subscale scores can also be obtained by summing responses to items in each of the three subscales. Possible scores on each subscale range from four to 28 while the full-scale score can range from 12 to 84. Higher scores indicate individuals with higher levels of perceived social support. The MSPSS is reliable and valid, with a strong internal consistency (α = 0.94–0.96).

#### Family hierarchy

The Chinese version of the Personal and Relationship Profile scale (PRP) (28, 29) was used to assess the three aspects of family hierarchy: male dominance, violence approval, and violent socialization. Male dominance was measured using the six-item Dominance subscale, violence approval with the 10-item Violence Approval PRP subscale, and violent socialization with the five-item Violent Socialization subscale. All items were rated on a four-point scale, ranging from strongly disagree to strongly agree, with higher scores indicating higher levels of family hierarchy. The Chinese version of the PRP scale has been translated and validated, demonstrating good psychometric properties (29). The subscales demonstrated satisfactory reliability in this study (α = 0.84–0.88). Pairwise correlations between the three PRP subscales ranged from 0.38 to 0.49, reflecting moderate intercorrelation among related psychological constructs.

#### Health - related quality of life and mental health symptoms

The Chinese version of the 12-item Short Form Health Survey (SF-12v2) (30) was used to assess health-related quality of life. Item scores were weighted and summarized into two components: the physical component score (PCS) and the mental component score (MCS), both ranging from 0 to 100. Higher scores indicated better health-related quality of life. The scale demonstrated satisfactory reliability (α = 0.88). The 21-item Chinese version of the Depression Anxiety Stress Scale (DASS-21) (31) was used to measure depression, anxiety, and stress levels in parents. Each item assessed the frequency of symptoms over the past 7 days and was rated on a four-point Likert scale, with excellent reliability (α = 0.87–0.91). Children's health-related quality of life was measured using the Chinese version of the Pediatric Quality of Life Inventory Generic Core Scale (PedsQoL) (32), covering physical, emotional, social, and school functioning, with strong reliability across age groups (α = 0.91–0.96).

### Data analysis

Individual and family-based victimization outcomes, including polyvictimization, were summarized using descriptive statistics. Prevalence estimates were reported for both lifetime and preceding-year experiences, and were further categorized by victimization targets and the number of victims within the household. To compare characteristics between families with and without parents with health conditions, independent-samples *t*-tests were used for continuous variables, and Chi-square tests were used for categorical variables.

To further examine how FPV and related characteristics differed between the two groups, we conducted logistic regression analyses in which the dependent variable was the presence of parents with health conditions (coded as 1 = disability/illness, 0 = non-disability/illness). Independent variables included demographic characteristics (e.g., age, gender, income), presence of care dependents (e.g., presence of children with special educational needs (SEN) or grandparents with disabilities or chronic illnesses), family hierarchy, social support, and FPV exposure. This modeling strategy was used solely to estimate odds ratios (ORs) and adjusted odds ratios (aORs) that describe how violence exposure differs between the two groups, rather than to imply any directional or causal relationship. Variables in phase 1 were adjusted for other variables in the same phase. Variables in phase 2 were adjusted for all variables in phases 1, and variables in phase 3 were adjusted for all variables in phases 1, 2, and 3. Covariates were selected based on theory and prior research to minimize confounding while avoiding overadjustment (i.e., table 2 fallacy) (33). Multicollinearity was assessed using variance inflation factors (VIF). All VIF values were well below the critical threshold of 5, indicating no problematic multicollinearity and confirming the stability of the regression models. *Post-hoc* power analysis was conducted using observed proportions and Cohen's h. Given the cross-sectional design, no assumptions were made regarding temporal ordering between disability/chronic illness and victimization, and all findings are interpreted as associational comparisons. All *p*-values were two-tailed, with *p*-values < 0.05 considered significant. Analyses were conducted using IBM SPSS Statistics for Windows, version 29.0 (IBM Corp., Armonk, NY, United States) (34).

## Results

## Socio-economic and health profiles of respondents

Descriptive statistics are reported in Table 1. Respondents with health conditions were generally older (*p* < 0.001), had lower education levels (*p* < 0.01) and a higher rate of unemployment (*p* < 0.01). They also reported lower average monthly household income (*p* < 0.05), and were more likely to receive CSSA (*p* < 0.001). 84.3% of respondents in the health condition group reported chronic illness (*p* < 0.001). 76.4% had one condition, 18.6% had two, and 5.0% had three. In terms of care dependents, households in the health condition group were significantly more likely to have children with special educational needs (*p* < 0.001), and grandparents with disability or chronic illness (*p* < 0.001). Respondents with health conditions also reported significantly higher violence approval scores (*p* < 0.001), male dominance scores (*p* < 0.001), and violence socialization scores (*p* < 0.001) than their non-disability group.

**Table 1:** Demographic characteristics of respondents and families.

Variable	Total (*N* = 1,400)	Health conditions (*N* = 140)	Non-health conditions (*N* = 1,260)	χ^2^/*t*
Respondent's gender				1.293
Male	24.6%	28.6%	24.2%	
Female	75.4%	71.4%	75.8%	
Respondent's age (mean, SD)	39.75 (6.99)	42.44 (8.14)	39.45 (6.79)	4.198^***^
Respondent' education level				19.140^**^
Primary or below	0.5%	2.1%	0.3%	
Secondary school	42.9%	45.7%	42.7%	
College	20.7%	21.4%	20.6%	
University	35.9%	30.7%	36.5%	
Respondent's unemployment	2.5%	6.4%	2.1%	9.849^**^
Marital status				0.528
Separated/divorced/widowed	6.4%	5%	6.6%	
Cohabited	93.5%	95%	93.4%	
Average monthly household income HK$ (mean, SD)	49,600 (24,275)	45,250 (24,889)	50,083 (24,169)	−2.238^*^
Respondent receives Comprehensive Social Security Assistance (CSSA)	2.2%	7.1%	1.7%	17.451^***^
Respondent's health conditions				
Physical disability		0.7%		
Visual impairment		2.1%		
Hearing impairment		2.1%		
Speech impairment		0.0%		
Mental illness		18.6%		
Intellectual disability		0.0%		
Autism		0.7%		
Attention-deficit/hyperactivity disorder (ADHD)		1.4%		
Specific Learning Difficulties (SpLD)		2.1%		
Chronic illness		84.3%		
Number of disabilities/Chronic illnesses				NA
0	90.0%	0.0%	100.0%	
1	7.6%	76.4%	0.0%	
2	1.9%	18.6%	0.0%	
3	0.5%	5.0%	0.0%	
Households with SEN children	9.3%	30.0%	7.0%	79.238^***^
Households with grandparents with disability and/or chronic illness	57.0%	84.3%	54.5%	16.924^***^
Violence Approval (mean, SD)	1.79 (0.47)	1.96 (0.44)	1.77 (0.47)	4.438^***^
Male dominance (mean, SD)	2.01 (0.55)	2.25 (0.47)	1.99 (0.55)	6.043^***^
Violence Socialization (mean, SD)	0.76 (0.66)	1.10 (0.71)	0.73 (0.64)	6.403^***^

χ^2^ = Chi-square test, *t* = *T*-test, ^*^p < 0.05, ^**^p < 0.01, ^***^p < 0.001.

Respondents with health conditions exhibited significantly poorer health and psychosocial outcomes compared to those without such conditions (Table 2). For overall health functioning, respondents with health conditions reported lower physical health (*p* < 0.001) and mental health (*p* < 0.001). Regarding mental health symptoms, respondents with health conditions reported significantly higher levels of depression (*p* < 0.001), anxiety (*p* < 0.001), and stress (*p* < 0.001). Perceived social support was also significantly lower in the health condition group compared to the non-health condition group (*p* < 0.001). Children's quality of life was significantly lower in households with a respondent with health conditions across multiple age groups. Significant differences were found for children aged 2–4 (*p* < 0.01), ages 5–7 (*p* < 0.01), and ages 8–12 (*p* < 0.001). The difference for adolescents aged 13–17 was not statistically significant.

**Table 2:** Comparison of health variables (health conditions vs. non-health conditions).

Variable	Mean (SD)			
	Total (***N*** = **1,400)**	**Health conditions (*****N*** = **140)**	**Non-health conditions (*****N*** = **1,260)**	***t***
Physical health by SF-12 (mean, SD)	51.24 (6.37)	46.85 (8.57)	51.73 (5.88)	−6.564^***^
Mental health by SF-12 (mean, SD)	47.15 (9.92)	42.63 (11.11)	47.66 (9.65)	−5.148^***^
Depression symptoms	2.93 (3.88)	4.56 (4.83)	2.75 (3.72)	4.290^***^
Anxiety symptoms	2.51 (3.34)	3.76 (4.03)	2.37 (3.23)	3.944^***^
Stress symptoms	4.06 (4.12)	5.98 (4.70)	3.84 (4.00)	5.175^***^
Social support	5.16 (1.09)	4.72 (1.25)	5.21 (1.06)	−4.479^***^
Children's quality of life				
Age 2 to 4	79.19 (14.08) (*n* = 371)	70.73 (16.11) (*n* = 24)	79.78 (13.77) (*n* = 347)	−3.080^**^
Age 5 to 7	77.01 (16.84) (*n* = 346)	69.68 (16.74) (*n* = 38)	77.92 (16.66) (*n* = 308)	−2.874^**^
Age 8 to 12	85.35 (14.48) (*n* = 487)	75.77 (17.86) (*n* = 58)	86.65 (13.47) (*n* = 429)	−4.471^***^
Age 13 to 17	88.37 (12.73) (*n* = 448)	85.59 (13.99) (*n* = 55)	88.76 (12.51) (*n* = 393)	−1.731

*t* = *T*-test, ^**^p < 0.01, ^***^p < 0.001.

## Prevalence of family victimization

The individual-based and family-based prevalence of family victimization was computed (Tables 3 and 4, respectively). Respondents with health conditions consistently reported higher levels of family victimization compared to non-health condition families, in both their lifetime and the preceding-year. For the individual-based computation of prevalence (Table 3), respondents with health conditions experienced significantly higher lifetime prevalence of any type of violence against adult parents (25.2% vs. 14.5%, *p* < 0.001), any type of violence against children (26.2% vs. 16.0%, *p* < 0.001) and any type of violence against older adults (39.1% vs. 12.3%, *p* < 0.001). Similar disparities were observed for preceding-year prevalence (all *p* < 0.01). For the family-based computation of prevalence (Table 4), respondents with health conditions were significantly more likely to report adult victimization (32.9% vs. 21.0%, *p* < 0.01), any child victimization (28.6% vs. 16.5%, *p* < 0.001), and any elder victimization (18.6% vs. 6.7%, *p* < 0.001). Rates of lifetime polyvictimization (17.9% vs. 8.7%, *p* < 0.001) and preceding-year polyvictimization (12.1% vs. 5.9%, *p* < 0.01) were also notably higher in the health condition group.

**Table 3:** Prevalence of family victimization (individual-based) by health conditions.

Variable	Prevalence (%)					
	Lifetime prevalence			**Preceding-year prevalence**		
	**Health Conditions**	**Non-Health Conditions**	χ^2^	**Health Conditions**	**Non-Health Conditions**	χ^2^
**Violence against adult parent (*****N*** = **2,696; H** = **270, NH** = **2,426)**						
Intimate partner violence (IPV)	17.8%	11.2%	10.013^**^	11.1%	6.0%	10.551^**^
In-law violence (IL)	7.8%	3.8%	9.894^**^	2.6%	2.2%	0.148
Adult child violence (ACV)	15.9%	7.9%	19.878^***^	3.7%	2.3%	1.838
Parent abuse (PA)	12.2%	4.1%	33.991^***^	9.6%	3.6%	22.098^***^
Relative abuse (RA)	4.1%	1.2%	14.53^***^	1.5%	0.4%	6.244^*^
Any type	25.2%	14.5%	21.055^***^	16.3%	8.5%	17.345^***^
**Violence against children (*****N*** = **1,895; H** = **195, NH** = **1,700)**						
Child maltreatment (CM)	25.6%	15.5%	13.123^***^	19.5%	12.1%	8.469^**^
Grandchild maltreatment (GCM)	8.2%	5.8%	1.741	4.6%	4.2%	0.083
Sibling abuse (SA)	10.3%	5.0%	9.235^**^	9.7%	4.3%	11.247^***^
Relatives child maltreatment (RCM)	3.1%	1.1%	5.698^*^	1.5%	0.7%	1.544
Any type	26.2%	16.0%	12.756^***^	20.0%	12.8%	7.838^**^
**Violence against older adults (*****N*** = **1,316; H** = **119, NH** = **1,206)**						
Elder maltreatment (EM)	20.9%	8.0%	20.136^***^	9.1%	3.9%	6.562^*^
Elder intimate partner violence (EIPV)	27.3%	9.0%	36.033^***^	21.8%	5.7%	39.771^***^
Grandparent abuse (PA)	4.5%	1.9%	3.370	3.6%	1.2%	4.055^*^
Relative elder maltreatment (EM)	13.6%	3.1%	29.666^***^	8.2%	1.2%	27.102^***^
Any type	39.1%	12.3%	58.438^***^	25.5%	8.1%	34.962^***^

H, health conditions; ND, non-health conditions, χ^2^ = Chi-square test, ^*^p < 0.05, ^**^p < 0.01, ^***^p < 0.001.

**Table 4:** Prevalence of family victimization (family-based, *N* = 1,400 families) by health conditions.

Variable	Prevalence (%)					
	**Lifetime prevalence**			**Preceding-year prevalence**		
	**Health conditions (*****N*** = **140)**	**Non-health conditions (*****N*** = **1,260)**	χ^2^	**Health conditions (*****N*** = **140)**	**Non-health conditions (*****N*** = **1,260)**	χ^2^
Any type of violence against adult parent	32.9%	21.0%	10.197^**^	21.4%	12.4%	8.953^**^
Any type of violence against children	28.6%	16.5%	12.580^***^	20.7%	12.9%	6.441^*^
Any type of violence against older adults	18.6%	6.7%	24.665^***^	9.3%	4.5%	6.015^*^
Family victimization			19.353^***^			11.418^**^
No victimization	56.4%	72.9%		70.7%	81.5%	
1–2 victims	25.7%	18.5%		17.1%	12.6%	
Polyvictims	17.9%	8.7%		12.1%	5.9%	

^*^p < 0.05, ^**^p < 0.01, ^***^p < 0.001.

## Multivariable logistic regression

Regression analyses examined factors associated with families with parents with health conditions, compared with families without health conditions. Results are summarized in Table 5, with ORs and aORs reported for both lifetime and past-year outcomes. In both crude and adjusted models, older age, lower household income, and receipt of CSSA were consistently associated with higher odds of reporting parents with health conditions. After adjusting for covariates, several additional factors remained independently associated with higher odds of health conditions, including having a child with SEN, living with a grandparent with a disability or chronic illness, lower perceived social support, higher violence socialization, and poorer physical functioning (SF-12 PCS). Elder abuse was also significantly associated with higher odds of health conditions in the adjusted model. Overall, lifetime associations were broadly consistent with those observed for past-year estimates.

**Table 5:** Multivariable logistic regression of factors associated with parental health conditions.

Variable	Crude OR (95% CI)	aOR (95% CI)	Crude OR (95% CI)	aOR (95% CI)
	Lifetime prevalence	Lifetime prevalence	Preceding-year prevalence	Preceding-year prevalence
**Phase 1** ^a^				
Gender (respondent) Male vs. Female	1.252 (0.849, 1.847)	0.953 (0.607, 1.495)	1.252 (0.849, 1.847)	0.953 (0.607, 1.495)
Age	1.059^***^ (1.034, 1.084)	1.061^***^ (1.033, 1.089)	1.059^***^ (1.034, 1.084)	1.061^***^ (1.033, 1.089)
Education	1.250 (0.880, 1.775)	0.851 (0.564, 1.284)	1.250 (0.880, 1.775)	0.851 (0.564, 1.284)
Unemployed	2.317^*^ (1.201, 4.470)	1.531 (0.736, 3.187)	2.317^*^ (1.201, 4.470)	1.531 (0.736, 3.187)
Separated/divorced/widowed	0.746 (0.338, 1.648)	0.447 (0.187, 1.066)	0.746 (0.338, 1.648)	0.447 (0.187, 1.066)
Monthly household income	2.045^***^ (1.396, 2.995)	1.709^*^ (1.078, 2.709)	2.045^***^ (1.396, 2.995)	1.709^*^ (1.078, 2.709)
Households with CSSA	4.538^***^ (2.092, 9.845)	3.319^**^ (1.351, 8.151)	4.538^***^ (2.092, 9.845)	3.319^**^ (1.351, 8.151)
Households with SEN children	5.029^***^ (3.252, 7.778)	3.806^***^ (2.354, 6.156)	5.029^***^ (3.252, 7.778)	3.806^***^ (2.354, 6.156)
Households with grandparents with disability and/or chronic illness	1.406 (0.960, 2.060)	1.705^*^ (1.125, 2.584)	1.406 (0.960, 2.060)	1.705^*^ (1.125, 2.584)
Violence approval	2.253^***^ (1.564, 3.244)	1.260 (0.803, 1.979)	2.253^***^ (1.564, 3.244)	1.260 (0.803, 1.979)
Male dominance	2.513^***^ (1.775, 3.557)	1.510 (0.980, 2.327)	2.513^***^ (1.775, 3.557)	1.510 (0.980, 2.327)
Violence socialization	2.034^***^ (1.618, 2.558)	1.479^**^ (1.112, 1.969)	2.034^***^ (1.618, 2.558)	1.479^**^ (1.112, 1.969)
Social support (MSPSS)	0.694^***^ (0.600, 0.801)	0.831^*^ (0.703, 0.981)	0.694^***^ (0.600, 0.801)	0.831^*^ (0.703, 0.981)
**Phase 2** ^a^				
Any type of violence against adult parent	1.837^**^ (1.259, 2.681)	1.333 (0.866, 2.052)	1.930^**^ (1.247, 2.988)	1.179 (0.711, 1.954)
Any type of violence against children	2.023^***^ (1.362, 3.005)	1.185 (0.747, 1.878)	1.758^*^ (1.132, 2.732)	0.941 (0.560, 1.582)
Any type of violence against older adults	3.193^***^ (1.976, 5.160)	1.888^*^ (1.082, 3.294)	2.160^*^ (1.151, 4.055)	1.335 (0.656, 2.717)
**Phase 3** ^a^				
**Health impact of respondents and children**				
SF-12 (PCS)	0.903^***^ (0.881, 0.926)	0.927^***^ (0.899, 0.956)	0.903^***^ (0.881, 0.926)	0.928^***^ (0.900, 0.957)
SF-12 (MCS)	0.954^***^ (0.939, 0.970)	0.976 (0.951, 1.001)	0.954^***^ (0.939, 0.970)	0.976 (0.951, 1.001)
Depression symptoms	1.100^***^ (1.061, 1.142)	0.970 (0.877, 1.074)	1.100^***^ (1.061, 1.142)	0.969 (0.877, 1.072)
Anxiety symptoms	1.105^***^ (1.058, 1.153)	0.987 (0.893, 1.090)	1.105^***^ (1.058, 1.153)	0.979 (0.887, 1.082)
Stress symptoms	1.114^***^ (1.073, 1.156)	0.998 (0.906, 1.099)	1.114^***^ (1.073, 1.156)	1.008 (0.915, 1.110)
PedsQoL (children)	0.975^***^ (0.965, 0.985)	1.007 (0.992, 1.022)	0.975^***^ (0.965, 0.985)	1.006 (0.991, 1.021)

PCS, physical component score; MCS, mental component score; PedsQoL, pediatric quality of life inventory. OR, odds ratio, aOR, Adjusted odds ratio. For aOR, variables in phase 1 were adjusted by other variables in the phase 1. Variables in phase 2 were adjusted by all variables in the phase 1 only. Variables in phase 3 were adjusted by all variables in the phase 1 to 3. ^a^The dependent variable (parents with health conditions) is ordered: 0 = non-health conditions, 1 = health conditions. ^*^p < 0.05, ^**^p < 0.01, ^***^p < 0.001.

## Discussion

Rather than investigating a causal relationship between risk factors, FPV, and health conditions, the observed associations highlight a cluster of demographic, structural, relational, and health-related vulnerabilities that characterize families with parents with health conditions. These patterns help clarify the broader family contexts in which FPV is more likely to co-occur.

The strong and persistent associations with older age, lower household income, and reliance on CSSA suggest that families with parents with health conditions are embedded in structurally disadvantaged socioeconomic contexts. These conditions may constrain coping resources and amplify stress within households, therefore disrupting family functioning and increasing the likelihood that multiple forms of conflict and violence co-occur (34). Moreover, the strong associations with having children with SEN and co-residing with older family members with health conditions point to heightened caregiving demands within these households. When multiple family members require care, caregiving demands may accumulate across generations, increasing relational strain and reducing families' capacity to manage conflict and stress (36, 37). Perceived social support was also notably low in those families. Given that social support typically serves as a protective factor in high-stress contexts (38), its absence may intensify caregiving stress and limit coping capacities for families already grappling with economic hardship and health challenges (39, 40). Taken together, these findings suggest that parental health conditions rarely occur in isolation. Rather, they often co-occur with socioeconomic disadvantage and strained family relational climates, reflecting a broader pattern of interconnected vulnerabilities associated with family conflict, victimization, and helplessness.

After accounting for structural and caregiving-related factors, violence socialization remained independently associated with health condition-affected family status. This pattern is consistent with social learning theory and intergenerational transmission models, which propose that behavioral norms and psychological orientations are shaped through repeated socialization processes within families (41–43). Violence socialization reflects a deeper developmental process through which individuals internalize aggression as an acceptable response to conflict (44). Prolonged exposure to family environments in which violence is routinely modeled, tolerated, or left unchallenged may normalize aggressive behaviors and establish a foundation for later endorsement of violence approval and hierarchical gender norms (45). In this context, health conditions may coexist with family environments characterized by entrenched violence-related norms. Therefore, these findings underscore the importance of examining how relational socialization processes shape the lived experiences of individuals with health conditions and influence broader patterns of family functioning and vulnerability to violence.

Regarding violence, our analyses revealed higher prevalence rates of violence toward adults, children, and older adults in families with parents with health conditions, with polyvictimization notably more common. After adjusting for socioeconomic and structural covariates, older adults victimization remained statistically significant. This suggests that older adults-directed violence might reflect persistent relational dynamics embedded within familial histories, rather than emerging solely in response to immediate socioeconomic stressors. Moreover, families with parental health conditions were more likely to include older adults with chronic illness or disability. The presence of multiple family members with health conditions may increase caregiving demands and family stress, both of which have been identified as major risk factors for elder abuse (46, 47). Previous research also indicates that shared living environments significantly elevate elder abuse risk. Specifically, residing with multiple household members other than a spouse increases vulnerability to both financial (48) and physical abuse (49). In Hong Kong, high population density and limited living space often necessitate multigenerational cohabitation (50), potentially exacerbating interpersonal tensions and conflicts. Together, these factors may be associated with the higher prevalence of elder victimization observed in families with parental health conditions.

In contrast, partner and child victimization appeared more closely associated with contemporary socioeconomic stressors, including financial hardship, caregiving burdens, and limited social support. These differential patterns indicate that FPV in health condition-affected families reflect deeper, interconnected relational processes rather than simply accumulated individual victimization experiences. FPV is therefore best understood through a systemic relational lens, recognizing that distinct forms of violence originate from separate yet interrelated familial contexts. Effective FPV interventions thus require comprehensive, family-centered approaches that simultaneously address structural disadvantage, caregiving burdens, and enduring relational dynamics.

While mental health symptoms and children's quality of life were associated with health conditions at the bivariate level, these associations attenuated after accounting for socioeconomic and family-level factors. In contrast, poorer physical functioning remained independently associated, highlighting its central role in shaping daily family functioning. This indicates mental distress may stem more from broader structural and caregiving burdens than from disability alone (51, 52). Similarly, children's quality of life was also found to be lower in families affected by health conditions, particularly among younger age groups. Older children and adolescents may spend less time in family settings or benefit from protective external influences such as peer support and community resources, potentially buffering family-related stress (53). However, this association diminished after accounting for family-level factors, indicating that children's wellbeing may be more strongly influenced by the broader caregiving and relational environment than by the presence of parents with health conditions *per se*. Thus, integrated care approaches addressing physical, psychological, social, and economic dimensions are therefore essential to support these families effectively.

## Limitations and future directions

This study is among the first to compare FPV and family life experiences between families with parental health conditions and those without. However, several limitations should be acknowledged. First, the cross-sectional design does not allow for causal inference. Although both lifetime and past-year victimization were assessed, the study did not collect information on the onset, duration, or progression of the parental health condition. It is therefore not possible to determine whether family violence preceded, coincided with, or followed the onset of the condition. This temporal ambiguity limits the interpretation of pathways or mechanisms linking parental health conditions with FPV. Thus, the observed associations should be understood as patterns of co-occurring vulnerability rather than evidence of directional effects. Longitudinal studies that collect detailed information on the timing and course of both parental health conditions and victimization are needed to clarify temporal sequencing and examine potential mechanisms over time.

Second, the study relied on self-reported information from a single parent respondent in each household. This may have introduced biases such as social desirability, recall error, subjective interpretation, and proxy-reporting bias, particularly given the sensitive nature of family violence. In addition, because all study variables were obtained from the same respondent at a single time point, common method variance may have influenced the observed associations. Thus, reliance on a single informant is likely to have resulted in underestimation of the prevalence of victimization and FPV. Because the same reporting method was used for families with and without parental health conditions, similar levels of underreporting would be unlikely to eliminate the observed group pattern, although they could have affected the estimated magnitude of the differences. If reporting accuracy differed between groups, however, the association could have been either weakened or exaggerated. In addition, although the survey achieved a response rate of 64.8%, information on non-respondents was unavailable, precluding a formal assessment of non-response bias. Families experiencing more severe violence, poorer health, or greater socioeconomic disadvantage may have been less likely to participate, which could have further contributed to underestimation of the prevalence of victimization and FPV and may limit the generalizability of the findings. Therefore, the prevalence estimates should be interpreted as conservative, and the observed group differences and adjusted associations should be interpreted with caution. Additionally, the reported health conditions were not independently verified through clinical diagnoses or medical records, which may limit the clinical applicability of the findings. Future research should incorporate multiple family informants and complementary data sources, such as clinical assessments, administrative records, or observational methods, and appropriate approaches to assess and minimize common method variance.

Third, disability and chronic illness were combined into a composite category because the small number of parents with disabilities did not permit reliable disaggregated analyses. In particular, chronic illness accounted for 84.3% of the parental health-condition group, suggesting that the observed associations may primarily reflect the experiences of families affected by parental chronic illness rather than those of parents with disabilities. Therefore, the findings should not be generalized to all parents with disabilities or interpreted as demonstrating that disability and chronic illness have equivalent associations with FPV. Future studies should recruit sufficiently large samples to distinguish disability from chronic illness and examine differences according to condition type, severity, duration, functional limitation, and associated caregiving needs.

## Conclusion

Taken together, these findings suggest that families with parents with health conditions are embedded in family contexts marked by structural disadvantage, dense caregiving demands, limited social support, and entrenched relational norms. Together, these conditions may be associated with family environments in which FPV is more likely to co-occur. By comparing families with vs. without parents with health conditions, this study demonstrates that health condition-affected households face a distinct constellation of vulnerabilities that extend beyond individual health conditions. Importantly, because these families are often more visible to health, disability, and caregiving service systems, they may represent a critical and feasible entry point for early FPV identification and screening. Integrating family-level violence screening into disability- and caregiving-related services may help disrupt cycles of violence and vulnerability, supporting the development of more effective family-centered prevention and intervention strategies.

## Data Availability

The raw data supporting the conclusions of this article will be made available by the authors, without undue reservation.
